# Multiple-Gene Regulation for Enhanced Antitumor Efficacy with Branch-PCR-Assembled TP53 and MYC Gene Nanovector

**DOI:** 10.3390/molecules27206943

**Published:** 2022-10-16

**Authors:** Longhuai Cheng, Liqing Lu, Ziyi Chen, Dejun Ma, Zhen Xi

**Affiliations:** State Key Laboratory of Elemento-Organic Chemistry, Department of Chemical Biology, National Pesticide Engineering Research Center, Collaborative Innovation Center of Chemical Science and Engineering, College of Chemistry, Nankai University, Tianjin 300071, China

**Keywords:** branch-PCR, gene therapy, TP53, MYC, genome therapy, cancer therapy, network regulation

## Abstract

Multiple proteins are involved in network regulation through the crosstalk of different signaling pathways in cancers. Here, we propose a novel strategy of genome therapy with branch-PCR-assembled gene nanovectors to perform network-based gene regulation at multiple levels for cancer therapy. To validate network-based multiplex-gene regulation for genome therapy, we chose to simultaneously target one tumor suppressor gene (*TP53*) and one oncogene (*MYC*) in two different signaling pathways. The results showed that, compared to gene nanovectors targeting single genes (NP-TP53 and NP-shMYC), branch-PCR-assembled gene nanovectors simultaneously expressing p53 proteins and MYC shRNA arrays (NP-TP53-shMYC) showed enhanced antitumor efficacy in both MDA-MB-231 cancer cells and an MDA-MB-231-tumor-bearing mouse model. These findings indicate the feasibility and effectiveness of genome therapy in cancer therapy.

## 1. Introduction

According to the central dogma, chromatin DNA is spatiotemporally regulated at multiple levels, including chromatin unfolding, DNA transcription, post-transcription, mRNA translation and post-translation [1,2,3,4]. Accordingly, gene regulation tools at multiple levels have also been discovered and artificially exploited for different biological research and gene therapy applications [5,6,7]. With the deep knowledge of the evolution and progression of complex diseases such as cancers, single-target-based gene therapy has faced great challenges in reducing side effects and drug resistance [8,9,10]. The fast development of novel gene delivery methods and gene regulation technologies moved gene therapy from single-gene-causing illnesses to multiple-gene-associated disorders in a more personalized, precise, safe and efficient manner [11,12,13]. To find an efficient therapeutic solution, strategies for mimicking chromatin DNA to precisely regulate gene expression by combining various gene regulation tools at different levels as an integrative toolbox have been promising for combatting complex diseases in a near-natural way. In this way, a number of gene regulation tools could be rationally integrated as a smart toolbox and loaded into chromatin-like payloads to mimic the chromosome-mediated gene-decoding process for disease therapy. Therefore, here, we use the term genome therapy to describe this artificial chromosome-like gene network regulation at multiple levels with different tools simultaneously based on the central dogma.

If multiple-gene regulation tools at different levels are integrated as an efficient toolbox for combination therapy, the delivery of such a gene regulation toolbox is still the greatest barrier, which indicates that a proper method of delivering such a gene regulation toolbox into a single cell must be found to enable the realization of the concept of genome therapy. To date, the applications of the available combination strategy have been advancing slowly due to the lack of a suitable vector for genome therapy. For this kind of vector, the balance of gene-loading capacity and nanoparticle size is critical to ensure the high performance of the combination strategy [14,15]. 

For this purpose, branch-PCR, defined as branched-DNA-primer-mediated PCR, has been used to provide technical support to assemble long double-stranded DNA (as gene modules) into nanostructured DNA for gene regulation, such as gene expression, gene silencing and genome editing [16,17,18,19,20,21,22]. Compared with the traditionally used non-viral vectors (linear DNA and plasmids) and viral vectors, branch-PCR-assembled gene nanovectors have a nanoscale size and a high percentage of gene content with high stability against nuclease and serum [22]. Furthermore, this nanovector also shows low cytotoxicity and immunogenicity due to its lack of bacterial sequences, unmethylated CpG motifs and bacterial endotoxin contaminants [23]. More importantly, this gene nanovector is suitable for genome therapy since it can carry diverse gene regulation toolboxes together as an all-in-one nanovector to spatiotemporally mediate network regulation at multiple levels. These characteristics not only make it applicable to gene therapy against single-gene-related diseases but also expand it to genome therapy against multiple-gene-associated diseases. 

As is known, in a wide range of malignant cancers, multiple hub proteins are involved in network regulation through the crosstalk of different signaling pathways [24,25]. For instance, there is a common feature in the dysregulation of tumor suppressor genes (TSR) and oncogenes [26]. As a result, it is difficult for single-gene-based therapy to both eliminate tumors and avoid relapses when only targeting one gene. Here, we propose the use of a branch-PCR-assembled gene nanovector to perform network-based multiplex gene regulation (combining gene overexpression and gene silencing) for genome therapy, hoping to not only achieve high therapeutic efficacy but also minimize side effects and recurrence as far as possible. To validate this network-based multiplex gene regulation for genome therapy, we chose to simultaneously target one tumor suppressor gene (TP53) and one oncogene (MYC) in two different signaling pathways that are tightly associated with cell proliferation and cell apoptosis. Normally, the p53 protein can repress the MYC promoter through histone deacetylation, and c-Myc can induce ARF transcription to inhibit the negative regulator of p53 (Mdm2) [27]. However, the abnormal overexpression of c-Myc and the loss of p53 in cancer cells enhance genomic instability and cause a high percentage of tumorigenesis [28]. Using a branch-PCR-assembled gene nanovector, the simultaneous recovery of the p53 protein and the reduction in the c-Myc protein will hopefully suppress tumor overgrowth in a more efficient manner.

**Figure 1:**
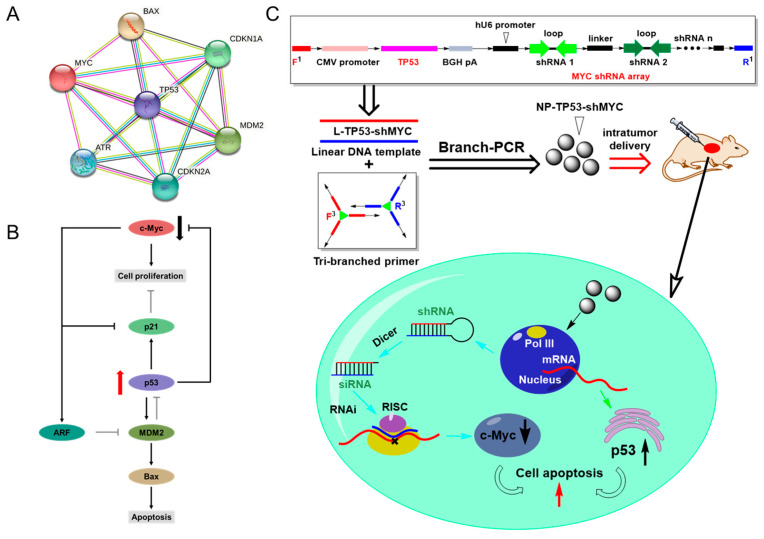
The network-based multiplex gene regulation of *TP53* and *MYC* for genome therapy using a branch-PCR-assembled nanovector. (**A**) An illustration of the gene network of cell proliferation and apoptosis. The interactive relationship of different proteins was constructed from the STRING database. (**B**) The dual targeting of hub genes for the network regulation of cell proliferation and apoptosis. (**C**) An illustration of the branch-PCR-assembled gene nanovector for genome therapy. A tri-branched primer pair (F^3^ and R^3^) was used to construct NP-TP53-shMYC nanovectors containing TP53 expression elements and MYC shRNA transcription elements. The nanovector could penetrate tumor cells to transcribe *TP53* mRNA and the *MYC* shRNA array. *TP53* mRNA was then translated into the p53 protein, and the *MYC* shRNA array was processed by Dicer into siRNAs to mediate the knockdown of *MYC* mRNA. The overexpression of p53 proteins and the elimination of c-Myc proteins suppressed tumor overgrowth.

In order to achieve synchronous TP53 gene overexpression and MYC gene knockdown, we used branch-PCR to assemble two gene expression cassettes of TP53 and a MYC shRNA array as an all-in-one gene nanovector. The in vitro and in vivo results showed that the simultaneous regulation of TP53 and MYC improved the antitumor efficacy compared to single-gene regulation. These findings demonstrate the feasibility and effectiveness of the concept of genome therapy in network regulation (Figure 1).

## 2. Results and Discussion

### 2.1. Formulation of NP-TP53-shMYC Nanovectors with Tri-Branched Primers

Tri-branched primers were successfully constructed according to previous methods [22]. Gene nanovectors were constructed from the gene cassette, which contained TP53 gene expression elements and MYC shRNA array transcription elements. The gene cassette (L-TP53-shMYC, 2932 bp) was arranged into four parts: (1) an F^1^ box and R^1^ box at both ends; (2) TP53 gene expression elements containing a CMV promoter, TP53 gene sequence and BGH terminator; (3) MYC shRNA array transcription elements containing hU6 and shRNA array expression cassettes targeting four sites of *MYC* mRNA; and (4) transcription terminator sequences (TTTTTT). In shRNA array expression cassettes, each shRNA unit consisted of the sense sequence, the loop sequence and the antisense sequence, and each shRNA unit was also spaced by 20 random nucleotides (linker) to facilitate the release of single shRNA by cellular Dicer nucleases. The plasmid DNA containing the gene cassette (P-TP53-shMYC) was constructed based on Golden gate assembly and confirmed by Sanger sequencing.

**Figure 2:**
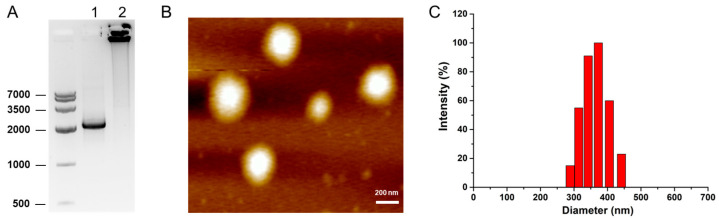
Construction and characterization of NP-TP53-shMYC. (**A**) Results of 1% agarose gel analysis for NP-TP53-shMYC. 1. L-TP53-shMYC; 2. NP-TP53-shMYC. (**B**) AFM analysis of NP-TP53-shMYC. Scale bar: 200 nm. (**C**) DLS analysis of NP-TP53-shMYC.

When P-TP53-shMYC was obtained, we used the linear primer pair to amplify the linear DNA (L-TP53-shMYC) based on the polymerase chain reaction (PCR). After gel purification and recovery, L-TP53-shMYC was used as a template for the second PCR with the above-mentioned tri-branched primers (F^3^ and R^3^). We called this branched-primer-constructed gene nanovector NP-TP53-shMYC. In contrast to L-TP53-shMYC, NP-TP53-shMYC showed remarkably slower mobility than linear DNA in 1% agarose gel (Figure 2A). This implies that branched primers participated in the assembly of linear gene cassettes into a structurally compact nanovector with a high molecular weight.

### 2.2. Characterization of NP-TP53-shMYC Nanovectors

To characterize NP-TP53-shMYC, we then performed atomic force microscopy (AFM) and dynamic light scattering (DLS). As shown in Figure 2B,C, NP-TP53-shMYC exhibited a condensed, clear and spherical nanostructure with a uniform diameter of 350 ± 30 nm. We then evaluated the serum stability of NP-TP53-shMYC by incubating it with 30% fetal bovine serum (FBS). The results showed that NP-TP53-shMYC could maintain structural stability in 30% FBS for 36 h, whereas P-TP53-shMYC and L-TP53-shMYC were almost completely degraded in 30% FBS in 2 h (Figure S1). This suggested that the branched-primer-formulated gene nanovector endowed this highly ordered compact structure with improved serum stability, which would be applicable to in vivo delivery.

### 2.3. NP-TP53-shMYC Simultaneously Expressed p53 Proteins and MYC shRNA Arrays in Breast Cancer Cells

To verify whether NP-TP53-shMYC could produce p53 proteins and reduce *MYC* mRNA in cancer cells, we chose a triple-negative breast cancer cell (MDA-MB-231) as the test cell since it has a low survival rate with strong invasiveness and still lacks an efficient therapeutic approach.

In the presence of Lipofectamine 2000, NP-TP53-shMYC was transfected into MDA-MB-231 cells at different concentrations (0.1–1.6 μg/mL). Meanwhile, a branch-PCR-constructed EGFP overexpression nanovector (NP-EGFP) was chosen as a negative control. At 48 h post-transfection, we evaluated the expression level of cellular *TP53* mRNA and *MYC* mRNA by RT-qPCR and also quantified the amount of p53 protein and c-Myc protein by Western blot.

**Figure 3:**
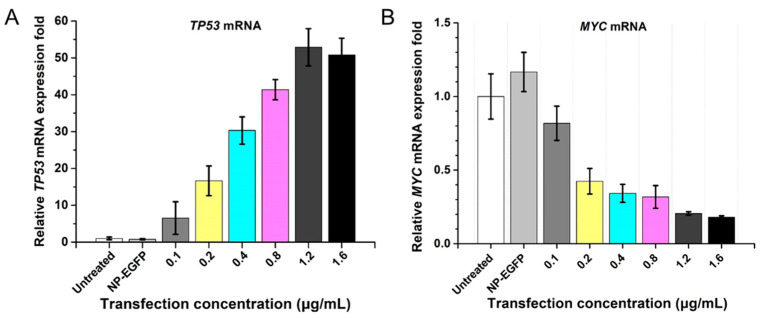
NP-TP53-shMYC induced simultaneous TP53 overexpression and MYC gene knockdown in breast cancer cells. (**A**) The histogram of the relative expression fold of *TP53* mRNA; (**B**) the histogram of the relative expression fold of *MYC* mRNA. The relative expression fold was quantified by RT-qPCR. NP-TP53-shMYC nanovectors at different concentrations were transfected into MDA-MB-231 cells for 48 h.

When NP-TP53-shMYC was transfected into MDA-MB-231 cells, we could observe a gradual increase in *TP53* mRNA and a rapid decrease in *MYC* mRNA as the transfection concentration of NP-TP53-shMYC increased (Figure 3A,B), which was also further confirmed by Western blot (Figure S2). When the transfection concentration of NP-TP53-shMYC reached 1.6 μg/mL, compared to the untreated group, the expression of *TP53* mRNA increased by 50 times, and *MYC* mRNA was accordingly decreased by 81.9%. As previously reported, NP-TP53 can increase *TP53* mRNA expression by 13 times [18], which is lower than the fold increase by NP-TP53-shMYC. The insertion of the shRNA array transcription cassette at the end of the TP53 gene expression cassette might enhance *TP53* mRNA transcription. These results demonstrated that NP-TP53-shMYC could be efficiently transfected into MDA-MB-231 cancer cells to co-transcribe *TP53* mRNA and the *MYC* shRNA array under separate promoters for multiplex gene regulation (simultaneous tumor suppressor gene rescue and RNAi-based oncogene silencing).

### 2.4. NP-TP53-shMYC Enhanced Breast Cancer Cell Apoptosis

Since the TP53 gene and MYC gene could be simultaneously regulated with NP-TP53-shMYC, we further investigated whether the dual targeting of the TP53 gene and MYC gene could lead to an enhancement of breast cancer cell suppression compared to single-gene targeting (NP-TP53 and NP-shMYC). After NP-TP53-shMYC at different concentrations (0–2.0 μg/mL) was transfected into MDA-MB-231 cells for 48 h with the help of Lipofectamine 2000, a cell apoptosis assay was performed by flow cytometry with FITC-labeled Annexin V and PI.

**Figure 4:**
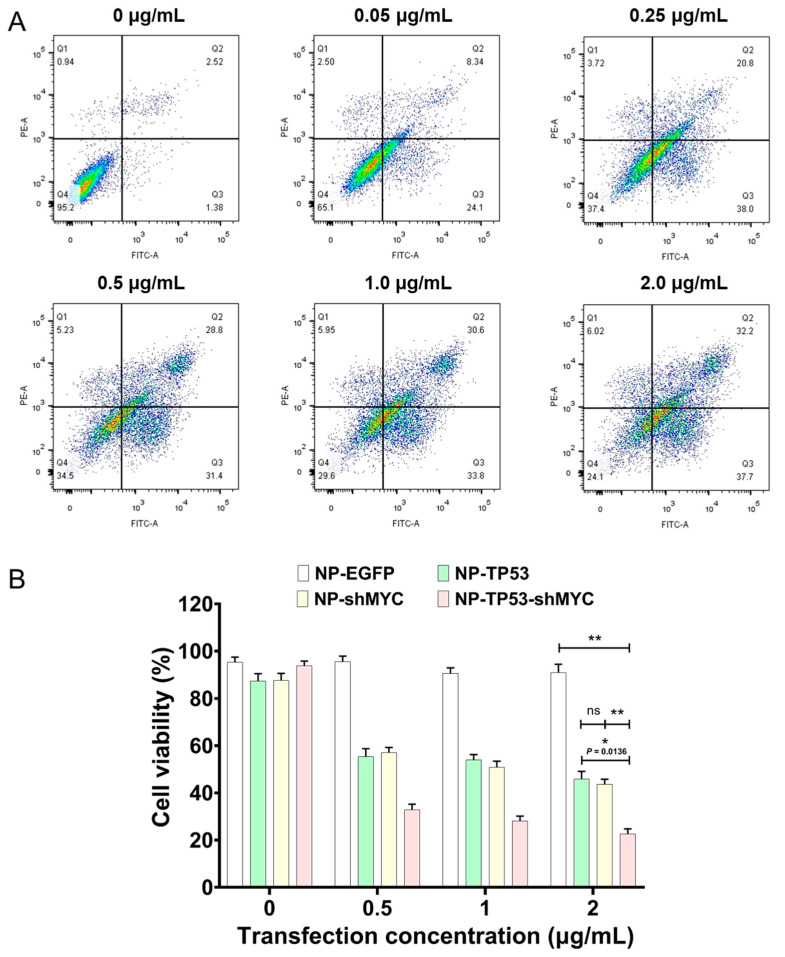
NP-TP53-shMYC enhanced breast cancer cell apoptosis. (**A**) A flow-cytometry-based assay of apoptosis with FITC-labeled Annexin V and PI was performed when MDA-MB-231 cells were transfected with different concentrations of NP-TP53-shMYC (0–2.0 μg/mL) by 1% Lipofectamine 2000 for 48 h. (**B**) Cell viability assay based on flow cytometry. The asterisk indicates a statistically significant difference of two different groups by two-sided Student’s *t*-test (ns, not statistically significant, * *p* < 0.05, ** *p* < 0.01).

The negative control (NP-EGFP) did not induce apoptosis in MDA-MB-231 cells (Figure S3). On the contrary, NP-TP53-shMYC induced significant apoptosis in MDA-MB-231 cells in a dose-dependent manner. When the transfection concentration of NP-TP53-shMYC was 2.0 μg/mL, the average apoptosis rate of MDA-MB-231 cells nearly reached 77.4% (Q1+Q2+Q3) (Figure 4A,B). Compared with NP-TP53-shMYC, the average apoptosis rates of MDA-MB-231 cells induced by NP-TP53 and NP-shMYC were 54.1% and 56.3%, respectively (Figure S4). The improved cell apoptosis ratio of NP-TP53-shMYC indicated that the dual targeting of the TP53 gene and MYC gene induced stronger cancer cell apoptosis than single-gene targeting via the possible synergistic enhancement of c-Myc proteins and p53 proteins in regulating cell apoptosis.

### 2.5. NP-TP53-shMYC Improved In Vivo Antitumor Efficacy

In view of the enhancement of cancer cell apoptosis by the dual targeting of the *TP53* gene and *MYC* gene, we further investigated antitumor efficacy in vivo using this strategy. We first established the MDA-MB-231 tumor xenograft model in female 6–8-week-old BALB/c nude mice. In order to systemically evaluate therapeutic effects and biological safety, we administered MDA-MB-231-tumor-bearing mice with different vectors at 2.25 mg/kg doses through an intratumoral injection every 2 days for a total of three treatments. Seven groups were analyzed: the untreated group, L-TP53-shMYC (L-TP53-shMYC with Lipofectamine 2000), L-TP53-shMYC(-) (L-TP53-shMYC without Lipofectamine 2000), NP-TP53 (NP-TP53 formulated with Lipofectamine 2000), NP-shMYC (NP-shMYC formulated with Lipofectamine 2000), NP-TP53-shMYC (NP-TP53-shMYC formulated with Lipofectamine 2000) and NP-TP53-shMYC(-) (NP-TP53-shMYC without Lipofectamine 2000).

**Figure 5:**
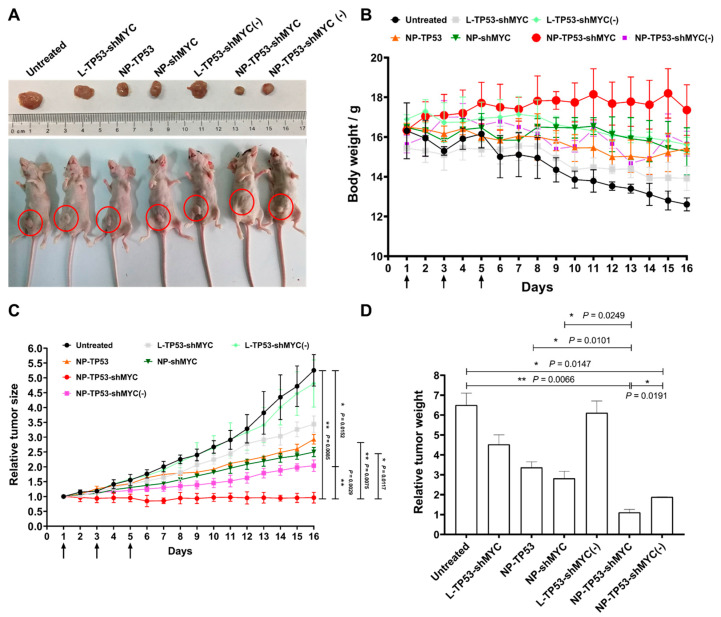
NP-TP53-shMYC inhibited in vivo tumor overgrowth through the simultaneous overexpression of p53 proteins and the strong elimination of c-Myc proteins. (**A**) Photograph of excised tumors and treated mice on the 16th day post-administration. (**B**) The change in the body weight of MDA-MB-231-tumor-bearing mice was recorded within 16 days after treatment in different groups (black arrows indicate time points of each treatment). (**C**) The change in the relative tumor volume of mice was recorded within 16 days after treatment in different groups (black arrows indicate the time points of each treatment). (**D**) Relative tumor weight in different treatment groups on the 16th day post-administration. Untreated: saline treatment; L-TP53-shMYC: L-TP53-shMYC formulated with Lipofectamine 2000; L-TP53-shMYC(-): L-TP53-shMYC without the help of Lipofectamine 2000; NP-TP53: gene nanovectors expressing *TP53* mRNA formulated with Lipofectamine 2000; NP-shMYC: gene nanovectors expressing *MYC* shRNA array formulated with Lipofectamine 2000; NP-TP53-shMYC: gene nanovectors expressing *TP53* mRNA and *MYC* shRNA array formulated with Lipofectamine 2000; NP-TP53-shMYC(-): NP-TP53-shMYC without the help of Lipofectamine 2000. Each MDA-MB-231-tumor-bearing mouse was administrated at 2.25 mg/kg doses through the intratumoral injection every 2 days for a total of three treatments (days 1, 3 and 5). The asterisk indicates a statistically significant difference of two different groups by two-sided Student’s *t*-test (* *p* < 0.05, ** *p* < 0.01).

Consistent with the in vitro anticancer effect, NP-TP53-shMYC showed better in vivo antitumor activity than all other treatments (Figure 5A). Compared to the untreated group, no significant changes in body weight were observed for any of the treatments (Figure 5B). In NP-TP53-shMYC-treated mice, the tumor volume did not greatly vary and was decreased by 85.2% compared to the untreated group on the 16th day post-administration (Figure 5C). The tumor weight was also reduced by 82.9% compared to the untreated group, which was much higher than that in all other treatments, especially NP-TP53 and NP-shMYC (Figure 5D). In NP-TP53-treated mice, the tumor volume and tumor weight were decreased by 47.9% and 48.2% compared to the untreated group on the 16th day post-administration (Figure 5C,D). In NP-shMYC-treated mice, the tumor volume and tumor weight were decreased by 57.6% and 56.7% compared to the untreated group on the 16th day post-administration (Figure 5C,D).

In contrast, L-TP53-shMYC(-) did not decrease the tumor volume or tumor weight. When Lipofectamine 2000 was added, L-TP53-shMYC showed improved antitumor activity, but it was still much lower than that obtained with NP-TP53-shMYC (Figure 5C,D). This result indicated that the linear-DNA-based cancer therapy seriously relied on the transfection agent for the lower nuclease resistance. We also noted that NP-TP53-shMYC(-) exhibited lower antitumor activity than NP-TP53-shMYC but showed higher antitumor activity than NP-TP53 and NP-shMYC (Figure 5C,D). This suggested that the branched-primer-assembled gene nanovector could also be effectively engulfed by solid tumors without the help of the transfection agent owing to its highly ordered nanoscale size for cell penetration and better nuclease resistance.

The in vivo antitumor tests revealed that tumor growth inhibition could be further improved when TP53 and MYC were both targeted. In contrast to gene nanovectors targeting a single gene (NP-TP53 or NP-shMYC), NP-TP53-shMYC could completely inhibit tumor growth. To verify the combinatory effect on p53 proteins and c-Myc proteins, we evaluated the mRNA and protein levels of TP53 and MYC in the tumor tissues by RT-qPCR and Western blotting. The results further proved that the better antitumor effect of NP-TP53-shMYC compared to that of NP-TP53 and NP-shMYC could be mainly attributed to simultaneous TP53 gene overexpression and MYC gene silencing in tumor tissues (Figures S5 and S6).

We subsequently determined the potential cytotoxicity and immune toxicity of gene nanovectors. The histopathological staining (H&E) analysis showed no obvious abnormalities, degenerations or lesions in the major organs, which also indicated that the injection of the gene nanovectors did not result in systemic toxicity (Figure S7). Next, we determined the levels of interleukin-6 (IL-6), tumor necrosis factor-α (TNF-α) and interferon-γ (IFN-γ) to estimate the immune-stimulatory effects. We found no significant increase in IL-6, TNF-α or IFN-γ levels in the serum for all treatments, suggesting that these gene nanovectors had no detectable immune-stimulatory adverse effects (Figure S8).

These results demonstrated that NP-TP53-shMYC could inhibit tumor overgrowth with no significant adverse effects through the simultaneous expression of p53 proteins and the strong knockdown of c-Myc proteins.

## 3. Materials and Methods

### 3.1. Chemical Synthesis of Branched Primers F^3^ and R^3^

The synthesis and characterization of the crosslinking molecule 3DBCO were performed using a previously established method [22]. In short, 5′ terminal azide-modified oligomers (F_N3_ or R_N3_) and unmodified oligonucleotides were synthesized from Sangon Biotech (Shanghai, China). The 5′ terminal azide-modified oligomer F_N3_ or R_N3_ (66 μg) was dissolved in ddH_2_O (57 μL), and then the crosslinking molecule 3DBCO (3.3 equiv) in DMF and PBS (20 μL) was added. The reaction mixture was gently stirred at 37 °C for 6 h. Finally, the tri-branched primers (F^3^ or R^3^) were purified by 8% denaturing PAGE containing 7 M urea.

### 3.2. Preparation of Linear DNA

Plasmid DNA containing the EGFP gene expression cassette, TP53 gene expression cassette and MYC shRNA array expression cassette was used to obtain L-EGFP, L-TP53 and L-shMYC, respectively (Tables S1–S5). The fusion of TP53 and MYC shRNA expression cassettes (molar ratio: TP53–MYC shRNA = 1:2) was accomplished with the Gibson assembly. L-TP53-shMYC was then obtained with L-TP53-MYC-F and L-TP53-MYC-R (Table S4) in a PCR reaction with the linear primer. PCR components (25 μL volume): 50 ng plasmids, 0.4 μM forward primer and 0.4 μM reverse primer, and 1×Prime STAR Max premix. PCR procedure: 98 °C, 30 s − (98 °C, 10 s − 60 °C, 10 s − 72 °C, 60 s) × 30 cycles − 72 °C, 5 min − 4 °C, 1 h. All of these linear DNA templates were purified with Zymoclean Gel DNA Recovery Kit (Zymo Research, Irvine, CA, USA).

### 3.3. Construction of Gene Nanovectors through Branch-PCR

Gene nanovectors (NP-TP53-shMYC, NP-TP53, NP-shMYC and NP-EGFP) were assembled using branch-PCR from a linear DNA template. Branch-PCR components (25 μL volume) were set as follows: 50 ng linear DNA template, 0.25 μM tri-branched primer pair (F^3^ and R^3^, Table S6) and 1×Prime STAR Max premix. PCR procedure: 98 °C, 30 s − (98 °C, 10 s − 60 °C, 10 s − 72 °C, 60 s) × 30 cycles − 72 °C, 5 min − 4 °C, 8 h. The branch-PCR-constructed gene nanovectors were purified with a GeneJET PCR Purification Kit (ThermoFisher Scientific, Irvine, CA, USA).

### 3.4. Characterization of NP-TP53-shMYC

AFM imaging: 3 μL of NP-TP53-shMYC (2.5 nM) was spotted on a freshly cleaved mica surface. After incubation for 5 min, 20 μL of Mg(OAc)_2_ (2 mM) was used to wash the sample one time. The sample was then dried under vacuum at room temperature before imaging. The sample was tested at room temperature in the tapping mode of a Nanoscope IV Atomic Force Microscope (Veeco Instruments Inc., Plainview, NY, USA).

DLS analysis: 0.25 μM NP-TP53-shMYC was used for DLS analysis with a laser wavelength of λ = 678 nm (scattering angle 908) at room temperature using a BI-200SM Particle Size Analyzer (Brookhaven Instruments Corp., Holtsville, NY, USA).

Serum stability: 5 μg of DNA (P-TP53-shMYC, L-TP53-shMYC, NP-TP53-shMYC) was incubated at 37 °C in 30% fetal bovine serum (FBS) with Mg(OAc)_2_ (0.25 μM) in a 50 μL reaction. Aliquots (5 μL) were taken at different times (0, 0.25, 0.5, 0.75, 1, 2, 4, 6, 8, 12, 24, 36, 48 and 96 h) and stored at −80 °C after adding 5 μL of glycerine to preserve the structures and inactivate the enzymes in FBS. The samples were analyzed on a 1% agarose gel.

### 3.5. Cell Culture and Transfection

Human breast cancer cells (MDA-MB-231) were cultured at 37 °C in a 5% CO_2_ atmosphere in high-glucose Dulbecco’s Modified Eagle’s Medium (DMEM, GIBCO) supplemented with 10% heat-inactivated fetal bovine serum (FBS, GIBCO), 100 U/mL penicillin, 4 mM L-glutamine and 100 μg/mL streptomycin (GIBCO) in the incubator. Before transfection, the culture medium was replaced with Opti-MEM (0.5 mL/well, GIBCO). The cells were co-transfected with NP-TP53-shMYC at different concentrations (0, 0.1, 0.2, 0.4, 0.8, 1.2 and 1.6 μg/mL per well in a 24-well plate) with Lipofectamine 2000 (Invitrogen, Carlsbad, CA, USA). After 4 h, each well was then supplemented with 1 mL of fresh DMEM supplemented with 10% FBS and maintained for 48 h.

### 3.6. Quantitative Real-Time PCR

After 48 h, MDA-MB-231 cells were washed with 1×PBS buffer three times. The cells were then harvested and suspended in lysis buffer (100 μL per well). Total RNA was extracted by AllPure DNA/RNA/Protein Kit (CoWin Biosciences, Tianjin, China). cDNA was obtained with SuperQuick RT MasterMix (CoWin Biosciences, Tianjin, China). RT-qPCR was performed using Real SYBR Mixture (CoWin Biosciences) and RT-qPCR primers (AuGCT Bioscience) using a CFX96 real-time PCR system (Bio-RAD). RT-qPCR assays were performed with the cycle threshold (2^−∆∆Ct^) method.

RT-qPCR primers were designed as:

*TP53*-F: 5′-GTGGTAATCTACTGGGACGGA-3′
*TP53*-R: 5′-CTTTCTTGCGGAGATTCTCTTC-3′
*MYC*-F: 5′-TGGTCTTCCCCTACCCTCTCAA-3′
*MYC*-R: 5′-TCCGTCGAGGAGAGCAGAGAAT-3′
*GAPDH*-F: 5′-GCTCTCTGCTCCTCCTGTTC-3′
*GAPDH*-R: 5′-ACGACCAAATCCGTTGACTCCG-3′

### 3.7. Western Blotting

Total cell protein was extracted using the AllPure DNA/RNA/Protein Kit (CoWin Biosciences, China). After separating on a 10% SDS–PAGE gel, the protein samples were transferred to polyvinylidene fluoride (PVDF) membranes. PVDF membranes were then blocked in TBST buffer (1×TBST buffer: 1.5 M NaCl, 20 mM Tris-HCl, 0.05% Tween-20) containing 5% non-fat milk powder for 1 h. After washing with 1×TBST buffer, PVDF membranes were incubated with anti-p53 (1:1000, CoWin Biosciences) or anti-c-Myc (1:1000, CoWin Biosciences) mouse monoclonal antibodies or anti-GAPDH (1:1000, CoWin Biosciences) mouse monoclonal antibody overnight at 4 °C. After washing four times with 1×TBST buffer, the PVDF membranes were incubated with the HRP-conjugated secondary antibody: goat anti-Mouse lgG (1:10,000, CoWin Biosciences) for 1 h at room temperature. Finally, target proteins were detected with an eECL Western blot kit (CoWin Biosciences).

### 3.8. Flow Cytometry

MDA-MB-231 cells were seeded into 6-well plates at a density of 1.6 × 10^5^ cells/well for 24 h. Each well was transfected with different concentrations (0, 0.05, 0.25, 0.5, 1 and 2 μg/mL) of different nanovectors (NP-TP53-shMYC, NP-TP53, NP-EGFP or NP-shMYC) using Lipofectamine 2000 (Invitrogen, Carlsbad, CA, USA). After 48 h, the cells were washed and harvested with 1×PBS buffer. The cells were then suspended in Annexin V binding buffer and incubated with FITC-labeled Annexin V and propidium iodide (PI) at room temperature in the dark for 30 min. Finally, the cell samples were immediately analyzed by flow cytometry (BD, Franklin Lakes, NJ, USA).

### 3.9. In Vivo Antitumor Test in MDA-MB-231-Tumor-Bearing Mouse Model

Female BALB/c nude mice (18–20 g, 6–8 weeks old) were purchased from Beijing Vital River Laboratory Animal Technology Co., Ltd. (Beijing, China). The MDA-MB-231-tumor-bearing mouse model was established by injecting 5 × 10^6^ MDA-MB-231 cells in the left abdominal cavity of BALB/c nude mice. When the MDA-MB-231 tumor volume reached 100 mm^3^, mice were randomly divided into 7 groups (n = 3 per group). The mice were separately administered saline (untreated control), L-TP53-shMYC (formulated with Lipofectamine 2000), NP-TP53 (formulated with Lipofectamine 2000), NP-shMYC (formulated with Lipofectamine 2000), NP-TP53-shMYC (formulated with Lipofectamine 2000), L-TP53-shMYC(-) (without any transfection reagent) and NP-TP53-shMYC(-) (without any transfection reagent) at 2.25 mg/kg doses via intratumoral injection every 2 days for a total of three treatments.

The body weight and tumor size were measured using an electronic balance and digital vernier caliper every day after the first injection. The tumor volume was calculated as follows: tumor size was measured across its longest (a) and shortest (b) diameters, and the volume was calculated according to the formula V = 0.5ab^2^. After 16 days of treatment, mice were sacrificed to collect blood samples for ELISA assays. IL-6, TNF-α and IFN-γ were analyzed by ELISA kits (CCC, Houston, Tx, USA). The tumors were imaged and weighed. The organs and the tumor tissues were excised for histological examination by standard hematoxylin and eosin (H&E) staining. In addition, on the 7th day, the tumors were divided into two portions for RT-qPCR and Western blotting.

## 4. Conclusions

In most malignant cancers, the abnormal counteraction of tumor suppressor genes such as p53 and oncogenes such as c-Myc in the cancer signaling network is a common phenomenon. Fine-tuning cell proliferation and cell apoptosis by altering the abnormal counteraction between tumor suppressor genes and oncogenes would be a key switch in network regulation to control tumor growth. For this purpose, we propose the concept of genome therapy through network-based gene regulation at multiple levels, which was preliminarily attempted by branch-PCR-assembled gene nanovectors for cancer therapy.

In this way, we propose the use of branch-PCR-assembled gene nanovectors for genome therapy against cancers, which could simultaneously load multiple gene cassettes targeting both tumor suppressor genes and oncogenes for network gene regulation. The newly constructed gene nanovector NP-TP53-shMYC had a smaller size than NP-TP53 and NP-shMYC. The in vitro and in vivo test results also demonstrated the better antitumor activity of NP-TP53-shMYC compared to NP-TP53 and NP-shMYC without detectable adverse cytotoxicity or immunological toxicity. The p53 rescue and c-Myc repression contributed to more efficient tumor control, which completely restricted tumor growth compared with other nanovectors targeting single genes. Notably, although linear DNA contained TP53 gene cassettes and MYC shRNA transcription cassettes, it had quite a low antitumor efficiency because of its inability to penetrate cells due to its size and because of its weak nuclease resistance. Hence, branch-PCR-assembled gene nanovectors are compatible with the concept of genome therapy. When multiple hub genes are considered for combination therapy, this gene nanovector, combined with different emerging gene regulation tools, will hopefully help to achieve network regulation at multiple levels for genome therapy against other complex diseases in the future.

## Data Availability

The data presented in this work are available in the article and Supplementary Materials.

## Supplementary Materials

The following supporting information can be downloaded at: https://www.mdpi.com/article/10.3390/molecules27206943/s1. Table S1: Primer sequences of constructed P-TP53-shMYC plasmids; Table S2: Primer sequences of L-TP53; Table S3: Primer sequences of L-shMYC; Table S4: Primer sequences of L-TP53-shMYC; Table S5: DNA sequence of L-TP53-shMYC cassette; Table S6: DNA sequences of F^3^ and R^3^ primers; Figure S1: Serum stability assay; Figure S2: The expression of p53 proteins and c-Myc proteins was quantified by Western blotting; Figure S3: Flow cytometry assay of anticancer activity of NP-EGFP; Figure S4: Flow cytometry assay of anticancer activity of NP-TP53; Figure S5: Quantitative analysis of the relative expression fold of MYC mRNA and TP53 mRNA in the tumors at 7 days post-administration; Figure S6: Western blotting analysis of p53 protein and c-Myc protein of tumor tissues at 7 days post-administration; Figure S7. Histochemistry staining images of organs and tumor tissues from mice sacrificed on the 16th day after treatment with various nanovectors; Figure S8. Analysis of immune cytokines levels (IL-6, TNF-α and IFN-γ) in the serum from mice sacrificed on the 16th day after treatment with various nanovectors.

## Sample Availability

Samples are available from the authors.
