# Supplementary material for: Multiple-Gene Regulation for Enhanced Antitumor Efficacy with Branch-PCR-Assembled TP53 and MYC Gene Nanovector
Source: Molecules. 2022 Oct 16;27(20):6943. doi: 10.3390/molecules27206943 (PMC9609172; doi:10.3390/molecules27206943)
Supplement: Supplementary file 1 [file molecules-27-06943-s001.zip › molecules-1977205-supplementary.pdf]

Supporting Information

Multiple gene regulation for enhanced antitumor efficacy with branch-PCR assembled TP53 and MYC gene nanovector

Longhuai Cheng,[‡1] Liqing Lu,[‡1] Ziyi Chen,[1] Dejun Ma,[*1] and Zhen Xi[*1]

[1] State Key Laboratory of Elemento-Organic Chemistry, and Department of Chemical Biology, National Pesticide Engineering Research Center, Collaborative Innovation Center of Chemical Science and Engineering, College of Chemistry, Nankai University, Tianjin 300071, P. R. China.

*Correspondence: DM: madejun@nankai.edu.cn, ZX: zhenxi@nankai.edu.cn, Tel: +86-022-23504782.

‡ The authors contributed equally to this work.

E-mail: madejun@nankai.edu.cn, zhenxi@nankai.edu.cn

Table S1 Primer sequences of constructed P-TP53-shMYC plasmids

Name	Sequence (5′-3′)
F-TP53	GGCGGGTGTGGTGGTTACGCGCAGC
R-TP53	GCGCTTAATGCGCCGCTACAGGGCG
F-MYC-TP53	CGCCCTGTAGCGGCGCATTAAGCGCGAGGGCCTATTTCCCATGAT
R-MYC-TP53	GCTGCGCGTAACCACCACACCCGCCCTCGAGAAAAAAGGAAACGA

Table S2 Primer sequences of L-TP53

Name	Sequence (5′-3′)
L-TP53-F	CCTCCCAAGATGGCCCGATAAGCTACAACAAGGCAAGGCT
L-TP53-R	CCTCCTCTTCCCTGTCCAAAAGGAAAGGACAGTGGGAGTG

Table S3 Primer sequences of L-shMYC

Name	Sequence (5′-3′)
L-MYC-F	CCTCCCAAGATGGCCCGATAGAGGGCCTATTTCCCATGAT
L-MYC-R	CCTCCTCTTCCCTGTCCAAACTCGAGAAAAAAGGAAACGA

Table S4 Primer sequences of L-TP53-shMYC

Name	Sequence (5′-3′)
L-TP53-MYC-F	CCTCCCAAGATGGCCCGATAAGCTACAACAAGGCAAGGCT
L-TP53-MYC-R	CCTCCTCTTCCCTGTCCAAACTCGAGAAAAAAGGAAACGA

Table S5 DNA sequence of L-TP53-shMYC cassette

1	CCTCCCAAGA	TGGCCCGATA	AGCTACAACA	AGGCAAGGCT	TGACCGACAA	TTGCATGAAG
61	AATCTGCTTA	GGGTTAGGCG	TTTTGCGCTG	CTTCGCGATG	TACGGGCCAG	ATATACGCGT
121	TGACATTGAT	TATTGACTAG	TTATTAATAG	TAATCAATTA	CGGGGTCATT	AGTTCATAGC
181	CCATATATGG	AGTTCCGCGT	TACATAACTT	ACGGTAAATG	GCCCGCCTGG	CTGACCGCCC
241	AACGACCCCC	GCCCATTGAC	GTCAATAATG	ACGTATGTTC	CCATAGTAAC	GCCAATAGGG
301	ACTTTCCATT	GACGTCAATG	GGTGGAGTAT	TTACGGTAAA	CTGCCCACTT	GGCAGTACAT
361	CAAGTGTATC	ATATGCCAAG	TACGCCCCCT	ATTGACGTCA	ATGACGGTAA	ATGGCCCGCC
421	TGGCATTATG	CCCAGTACAT	GACCTTATGG	GACTTTCCTA	CTTGGCAGTA	CATCTACGTA
481	TTAGTCATCG	CTATTACCAT	GGTGATGCGG	TTTTGGCAGT	ACATCAATGG	GCGTGGATAG
541	CGGTTTGACT	CACGGGGATT	TCCAAGTCTC	CACCCCATTG	ACGTCAATGG	GAGTTTGTTT
601	TGGCACCAAA	ATCAACGGGA	CTTTCCAAAA	TGTCGTAACA	ACTCCGCCCC	ATTGACGCAA
661	ATGGGCGGTA	GGCGTGTACG	GTGGGAGGTC	TATATAAGCA	GAGCTCTCTG	GCTAACTAGA
721	GAACCCACTG	CTTACTGGCT	TATCGAAATT	AATACGACTC	ACTATAGGGA	GACCCAAGCT
781	TACCATGGCC	TACCCCTACG	ACGTGCCCGA	CTACGCCTCC	CTCGGATCCG	AGGAGCCGCA
841	GTCAGATCCT	AGCGTCGAGC	CCCCTCTGAG	TCAGGAAACA	TTTTCAGACC	TATGGAAACT
901	ACTTCCTGAA	AACAACGTTC	TGTCCCCCTT	GCCGTCCCAA	GCAATGGATG	ATTTGATGCT
961	GTCCCCGGAC	GATATTGAAC	AATGGTTCAC	TGAAGACCCA	GGTCCAGATG	AAGCTCCCAG
1021	AATGCCAGAG	GCTGCTCCCC	CCGTGGCCCC	TGCACCAGCA	GCTCCTACAC	CGGCGGCCCC
1081	TGCACCAGCC	CCCTCCTGGC	CCCTGTCATC	TTCTGTCCCT	TCCCAGAAAA	CCTACCAGGG
1141	CAGCTACGGT	TTCCGTCTGG	GCTTCTTGCA	TTCTGGGACA	GCCAAGTCTG	TGACTTGCAC
1201	GTACTCCCCT	GCCCTCAACA	AGATGTTTTG	CCAACTGGCC	AAGACCTGCC	CTGTGCAGCT
1261	GTGGGTTGAT	TCCACACCCC	CGCCCGGCAC	CCGCGTCCGC	GCCATGGCCA	TCTACAAGCA
1321	GTCACAGCAC	ATGACGGAGG	TTGTGAGGCG	CTGCCCCCAC	CATGAGCGCT	GCTCAGATAG
1381	CGATGGTCTG	GCCCCTCCTC	AGCATCTTAT	CCGAGTGGAA	GGAAATTTGC	GTGTGGAGTA
1441	TTTGGATGAC	AGAAACACTT	TTCGACATAG	TGTGGTGGTG	CCCTATGAGC	CGCCTGAGGT
1501	TGGCTCAGAC	TGTACCACCA	TCCACTACAA	CTACATGTGT	AACAGTTCCT	GCATGGGCGG
1561	CATGAACCGG	AGGCCCATCC	TCACCATCAT	CACACTGGAA	GACTCCAGTG	GTAATCTACT
1621	GGGACGGAAC	AGCTTTGAGG	TGCGTGTTTG	TGCCTGTCCT	GGGAGAGACC	GGCGCACAGA
1681	GGAAGAGAAT	CTCCGCAAGA	AAGGGGAGCC	TCACCACGAG	CTGCCCCCAG	GGAGCACTAA
1741	GCGAGCACTG	CCCAACAACA	CCAGCTCCTC	TCCCCAGCCA	AAGAAGAAAC	CACTGGATGG
1801	AGAATATTTC	ACCCTTCAGA	TCCGTGGGCG	TGAGCGCTTC	GAGATGTTCC	GAGAGCTGAA
1861	TGAGGCCTTG	GAACTCAAGG	ATGCCCAGGC	TGGGAAGGAG	CCAGGGGGGA	GCAGGGCTCA
1921	CTCCAGCCAC	CTGAAGTCCA	AAAAGGGTCA	GTCTACCTCC	CGCCATAAAA	AACTCATGTT
1981	CAAGACAGAA	GGGCCTGACT	CAGACTGAGA	ATTCTGCAGA	TATCCATCAC	ACTGGCGGCC
2041	GCTCGAGCAT	GCATCTAGAG	GGCCCTATTC	TATAGTGTCA	CCTAAATGCT	AGAGCTCGCT
2101	GATCAGCCTC	GACTGTGCCT	TCTAGTTGCC	AGCCATCTGT	TGTTTGCCCC	TCCCCCGTGC
2161	CTTCCTTGAC	CCTGGAAGGT	GCCACTCCCA	CTGTCCTTTC	CTAATAAAAT	GAGGAAATTG
2221	CATCGCATTG	TCTGAGTAGG	TGTCATTCTA	TTCTGGGGGG	TGGGGTGGGG	CAGGACAGCA
2281	AGGGGGAGGA	TTGGGAAGAC	AATAGCAGGC	ATGCTGGGGA	TGCGGTGGGC	TCTATGGCTT
2341	CTGAGGCGGA	AAGAACCAGC	TGGGGCTCTA	GGGGGTATCC	CCACGCGCCC	TGTAGCGGCG
2401	CATTAAGCGC	GAGGGCCTAT	TTCCCATGAT	TCCTTCATAT	TTGCATATAC	GATACAAGGC
2461	TGTTAGAGAG	ATAATTGGAA	TTAATTTGAC	TGTAAACACA	AAGATATTAG	TACAAAATAC

2521	GTGACGTAGA AAGTAATAAT TTCTTGGGTA GTTTGCAGTT TTAAAATTAT GTTTTAAAAT
2581	GGACTATCAT ATGCTTACCG TAACTTGAAA GTATTTCGAT TTCTTGGCTT TATATATCTT
2641	GTGGAAAGGA CGAAACACCG CTTCACCAAC AGGAACTATG CGAACATAGT TCCTGTTGGT
2701	GAAGCCACAG TTCGGTAAGG GAGAGAGAAT GTCAAGAGGC GAACACGAAT GTTCGCCTCT
2761	TGACATTCTC CACAGTTCGG TAAGGGAGAG CTCATTTCTG AAGAGGACTT CGAAAAGTCC
2821	TCTTCAGAAA TGAGCCACAG TTCGGTAAGG GAGAGGAAAC GACGAGAACA GTTGACGAAT
2881	CAACTGTTCT CGTCGTTTCC TTTTTTCTCG AGTTTGGACA GGGAAGAGGA GG

Table S6 DNA sequences of F³ and R³ primers

Name	Sequence(5′-3′)
F^3	CCTCCCAAGATGGCCCGATA
R^3	CCTCCTCTTCCCTGTCCAAA

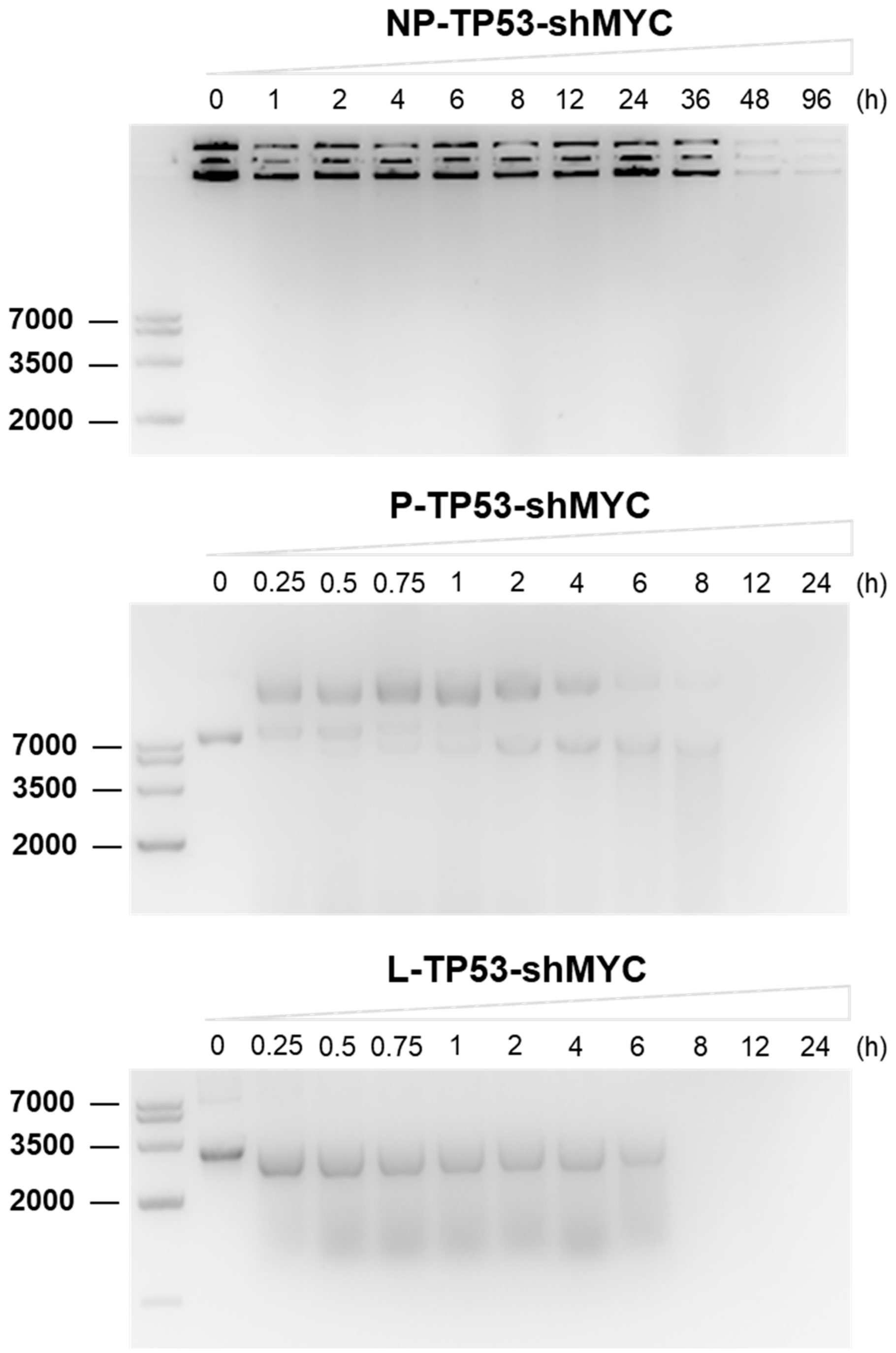

Figure S1. Serum stability assay. 5 μg DNA (NP-TP53-shMYC, P-TP53-shMYC, or L-TP53-shMYC) was incubated at 37℃ in 30% fetal bovine serum (FBS) with $Mg(OAc)_2$ (0.25 μM) in 50 μL reaction. Aliquots (5 μL) were taken at different time (0, 0.25, 0.5, 0.75, 1, 2, 4, 6, 8, 12, 24, 36, 48 and 96 h) and were analyzed by 1% agarose gel.

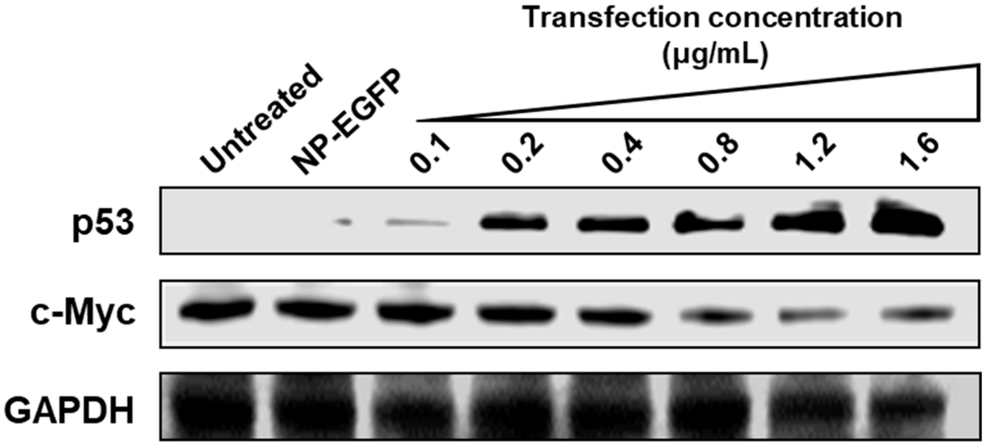

Figure S2. The expression of p53 proteins and c-Myc proteins was quantified by Western blotting. NP-TP53-shMYC nanovectors at different concentrations were transfected into MDA-MB-231 cells for 48 h.

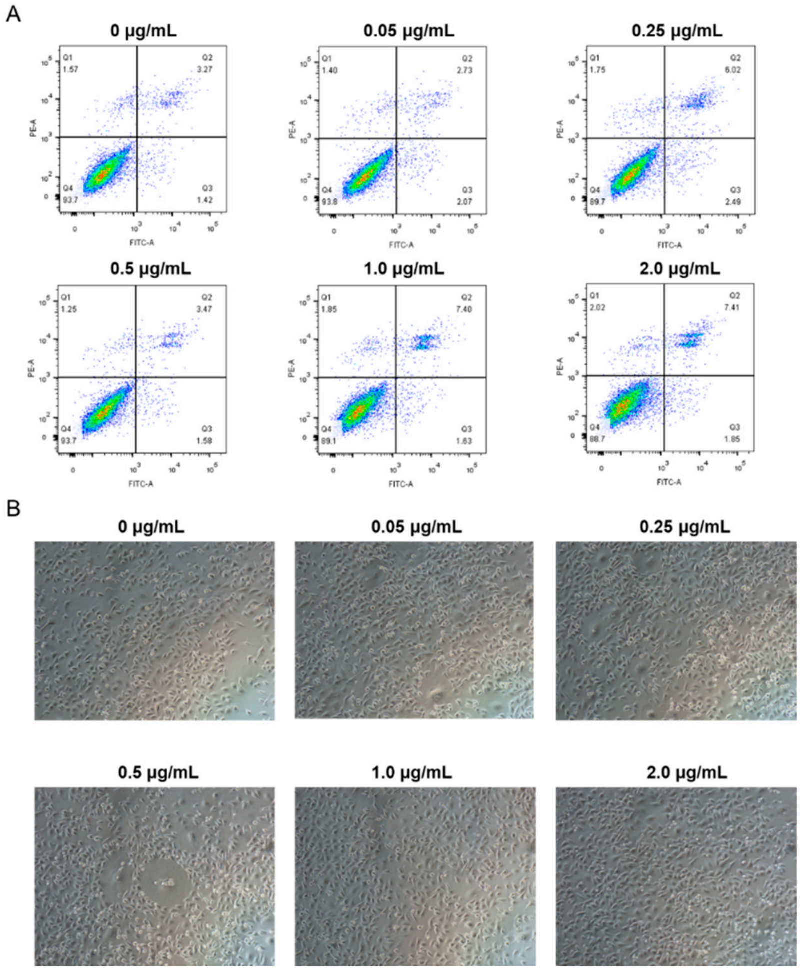

Figure S3. Flow cytometry assay of anti-cancer activity of NP-EGFP. (A) Dose-dependent apoptosis induction of MDA-MB-231 cells after transfection by 1% lipofectamine 2000 with different concentrations of NP-EGFP for 48 h. FITC-labeled Annexin V and PI were

used to discriminate the apoptosis; (B) The image of cell morphology at 48 h of post-transfection.

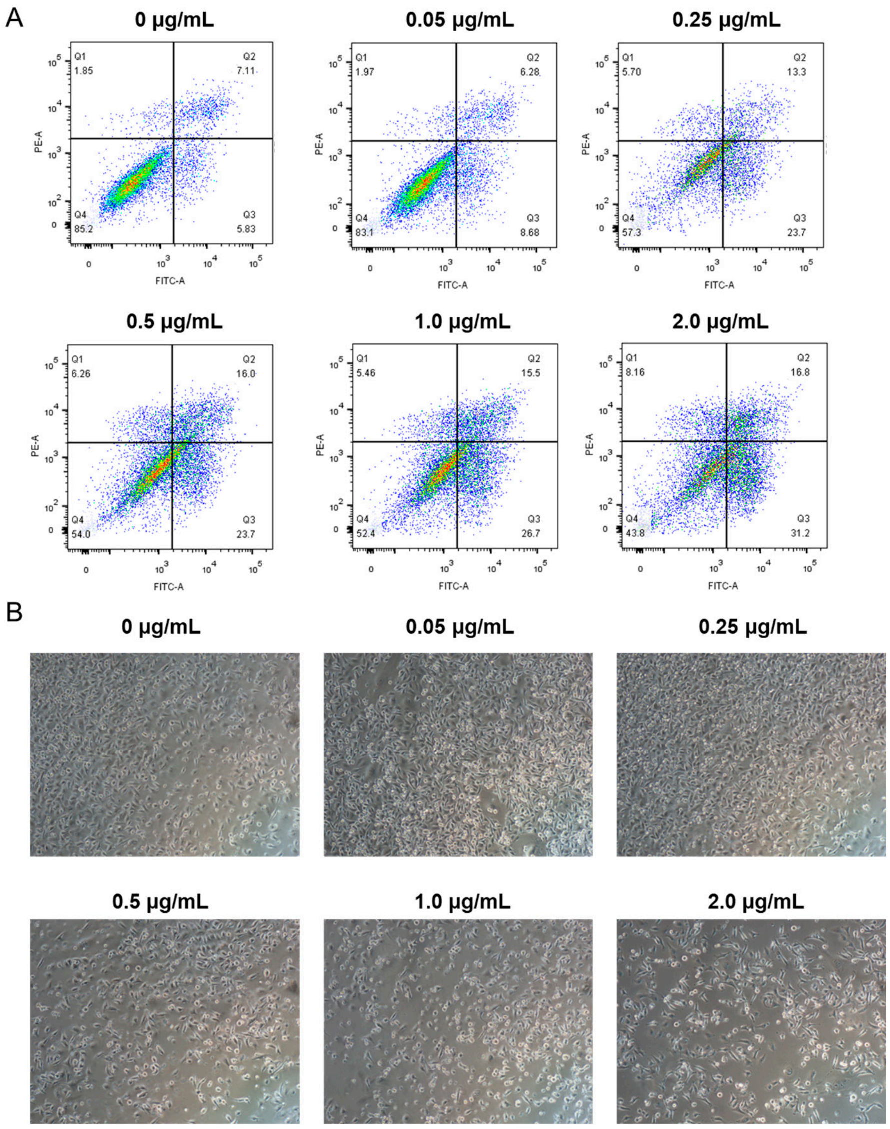

Figure S4. Flow cytometry assay of anti-cancer activity of NP-TP53. (A) Dose-dependent apoptosis induction of MDA-MB-231 cells after transfection by 1% lipofectamine 2000 with different concentrations of NP-TP53 for 48 h. FITC-labeled Annexin V and PI were used to discriminate the apoptosis; (B) The image of cell morphology at 48 h of post-transfection.

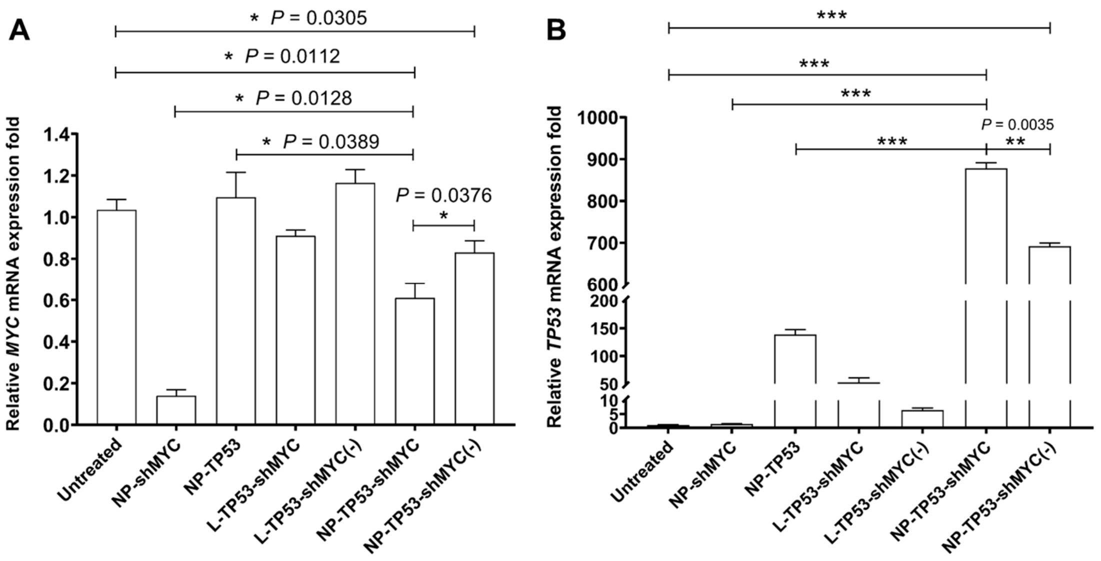

Figure S5. Quantitative analysis of the relative expression fold of *MYC* mRNA and *TP53* mRNA in the tumors at 7 days post-administration. A) the histogram of relative *MYC* mRNA expression fold; B) the histogram of relative *TP53* mRNA expression fold. Untreated: the saline treatment; L-TP53-shMYC: linear DNA formulated with Lipofectamine 2000; L-TP53-shMYC(-): L-TP53-shMYC without the help of Lipofectamine 2000; NP-TP53: gene nanovectors expressing TP53 formulated with Lipofectamine 2000; NP-shMYC: gene nanovectors expressing MYC shRNA array formulated with Lipofectamine 2000; NP-TP53-shMYC: gene nanovectors expressing TP53 and MYC shRNA array formulated with Lipofectamine 2000; NP-TP53-shMYC(-): NP-TP53-shMYC without the help of Lipofectamine 2000.

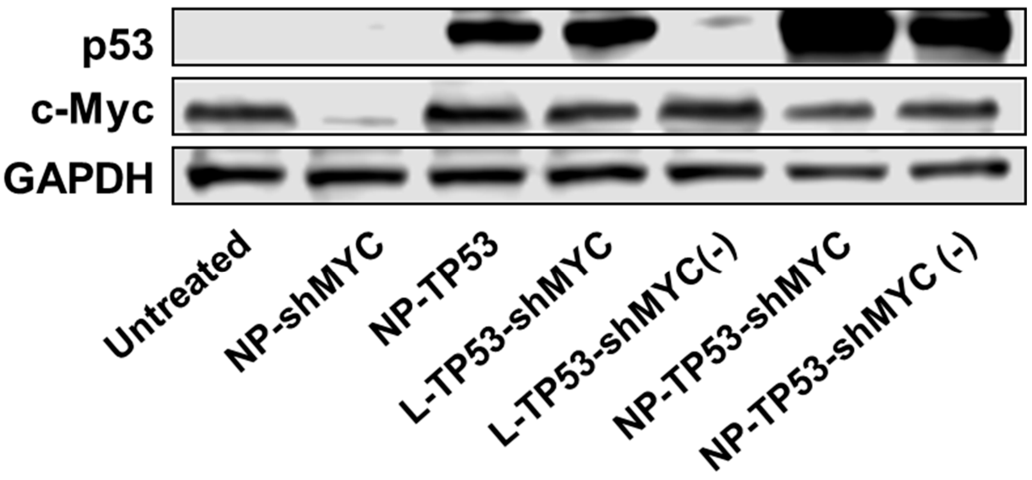

Figure S6. Western blotting analysis of p53 protein and c-Myc protein of tumor tissues at 7 days post-administration. Untreated: the saline treatment; L-TP53-shMYC: linear DNA formulated with Lipofectamine 2000; L-TP53-shMYC(-): L-TP53-shMYC without the help of Lipofectamine 2000; NP-TP53: gene nanovectors expressing TP53 formulated with Lipofectamine 2000; NP-shMYC: gene nanovectors expressing MYC shRNA array formulated with Lipofectamine 2000; NP-TP53-shMYC: gene nanovectors expressing TP53 and MYC shRNA array formulated with Lipofectamine 2000; NP-TP53-shMYC(-): NP-TP53-shMYC without the help of Lipofectamine 2000.

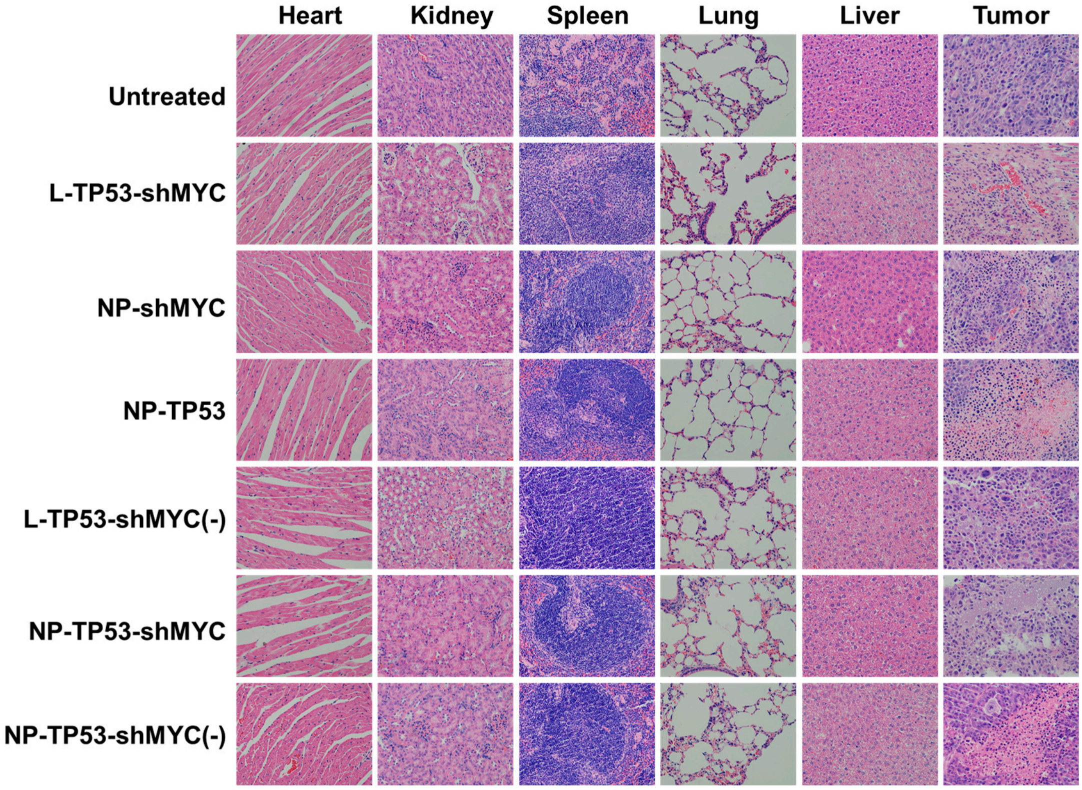

Figure S7. Histochemistry staining images of organs and tumor tissues from mice sacrificed on the 16th day after treatment with various nanovectors. Untreated: the saline treatment; L-TP53-shMYC: linear DNA formulated with Lipofectamine 2000; L-TP53-shMYC(-): L-TP53-shMYC without the help of Lipofectamine 2000; NP-TP53: gene nanovectors expressing TP53 formulated with Lipofectamine 2000; NP-shMYC: gene nanovectors expressing MYC shRNA array formulated with Lipofectamine 2000; NP-TP53-shMYC: gene nanovectors expressing TP53 and MYC shRNA array formulated with Lipofectamine 2000; NP-TP53-shMYC(-): NP-TP53-shMYC without the help of Lipofectamine 2000.

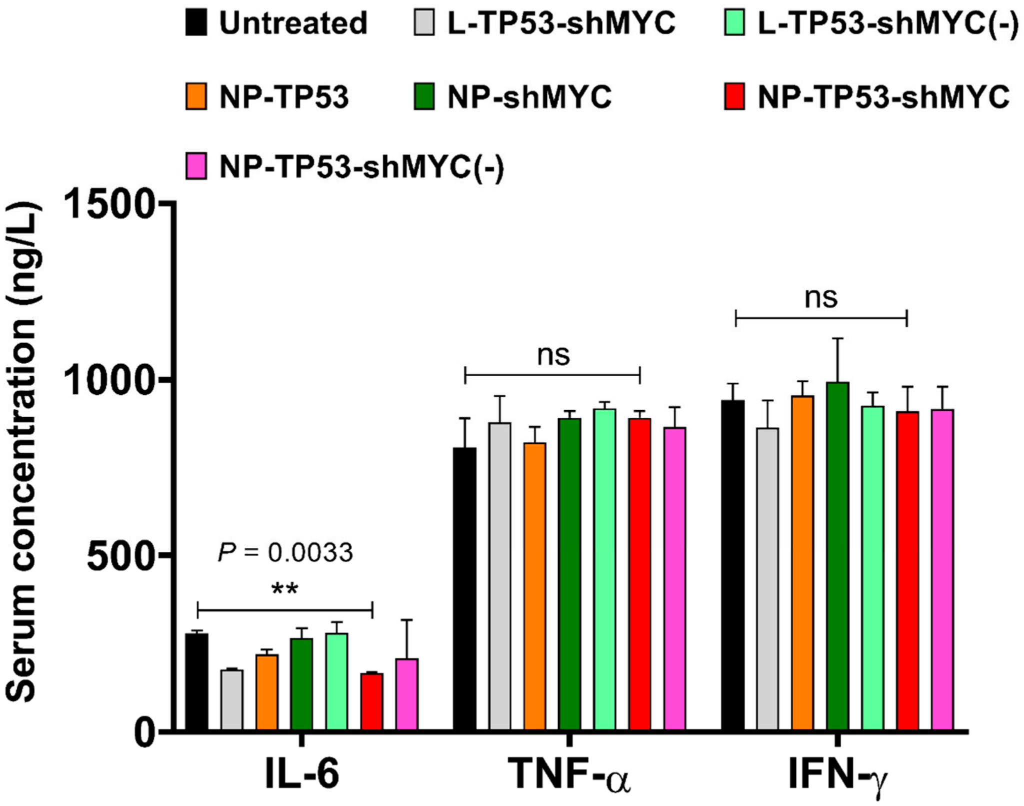

Figure S8. Analysis of immune cytokines levels (IL-6, TNF-α and IFN-ɤ) in the serum from mice sacrificed on the 16th day after treatment with various nanovectors. Untreated: the saline treatment; L-TP53-shMYC: linear DNA formulated with Lipofectamine 2000; L-TP53-shMYC(-): L-TP53-shMYC without the help of Lipofectamine 2000; NP-TP53: gene nanovectors expressing TP53 formulated with Lipofectamine 2000; NP-shMYC: gene nanovectors expressing MYC shRNA array formulated with Lipofectamine 2000; NP-TP53-shMYC: gene nanovectors expressing TP53 and MYC shRNA array formulated with Lipofectamine 2000; NP-TP53-shMYC(-): NP-TP53-shMYC without the help of Lipofectamine 2000.
